# Syrosingopine, an anti-hypertensive drug and lactate transporter (MCT1/4) inhibitor, activates hepatic stellate cells and exacerbates liver fibrosis in a mouse model

**DOI:** 10.1016/j.gendis.2023.101169

**Published:** 2023-11-18

**Authors:** Meichun Guo, Yannian Gou, Xiangyu Dong, Jiamin Zhong, Aohua Li, Ailing Hao, Tong-Chuan He, Jiaming Fan

**Affiliations:** a Ministry of Education Key Laboratory of Diagnostic Medicine, and the School of Laboratory Diagnostic Medicine, Chongqing Medical University, Chongqing 400016, China; b Molecular Oncology Laboratory, Department of Orthopaedic Surgery and Rehabilitation Medicine, The University of Chicago Medical Center, Chicago, IL 60637, USA

Syrosingopine is an anti-hypertensive drug and can cause high intracellular lactate levels and end-product inhibition of lactate dehydrogenase by inhibiting the lactate transporters MCT1 and MCT4. Previous studies have shown that syrosingopine plays an essential role in the process of glycolytic blockade, ATP depletion, and cell death in cancer due to high intracellular levels of lactate.^1^^,^^2^ The liver is the largest digestive gland in the human body and plays a crucial role in regulating energy metabolism, as well as serving as an important site for drug metabolism. Liver fibrosis is one of the most common pathological changes in the liver, which is a dynamic, highly complex molecular and cellular process leading to the excess accumulation of extracellular matrix components sustained by a heterogeneous population of hepatic myofibroblasts. Fibrosis usually follows a chronic and long-term liver injury, which may progress to hepatic cirrhosis, hepatocellular carcinoma, and liver failure.^3^ The major driver of liver fibrogenesis is the activated hepatic stellate cells (HSCs), which are also the major cellular sources of excessive matrix protein secretion.^4^ In this study, we investigated the effects of syrosingopine on HSCs in the progression of liver fibrosis.

The optimal concentration of syrosingopine was first determined by treating human immortalized hepatic stellate (LX2) cells, and 10 μM syrosingopine was used for further studies below (Fig. 1A). Syrosingopine was shown to significantly increase the lactate level in LX2 cells (Fig. 1B). Furthermore, the treated LX2 cells formed numerous dendrites, indicating that the HSCs were activated and transformed into fibroblast-like cells (Fig. 1C, panel a). During the process of HSC activation, intracellular energy homeostasis is dysregulated, which is manifested by autophagy and endoplasmic reticulum stress.^4^ We found in the LX2 cells treated with syrosingopine, the endoplasmic reticulum pool was slightly expanded, and protein synthesis decreased less, while the increased autophagy was apparent under transmission electron microscopy (Fig. 1C, panel b).

**Figure 1:**
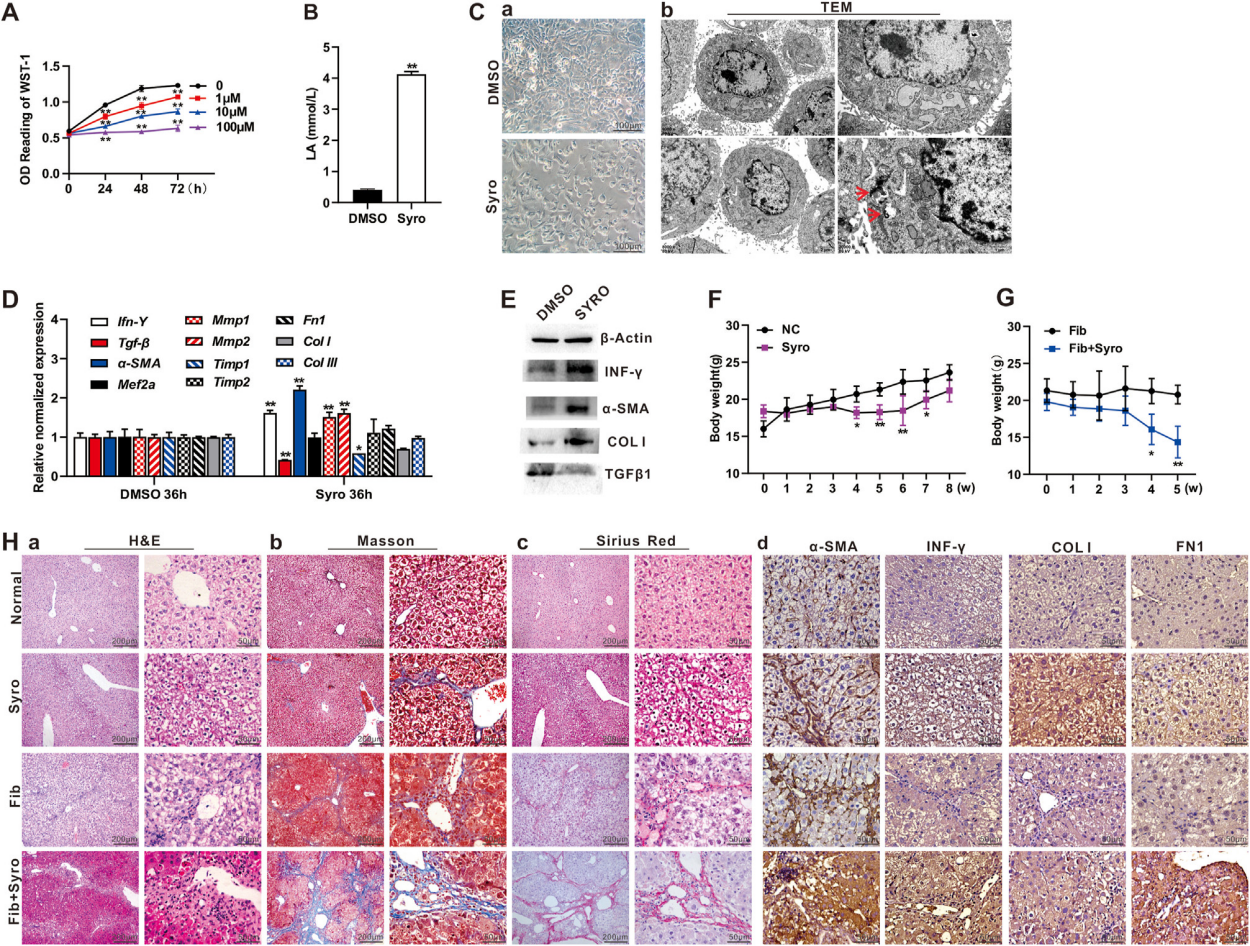
Syrosingopine activates hepatic stellate cells and exacerbates liver fibrosis by up-regulating the expression of α-SMA and INF-γ. **(A)** The optimal inhibitory concentration of syrosingopine in LX2 cells. LX2 cells were seeded in 96-well cell plates and treated with 0 μM, 1 μM, 10 μM, and 100 μM syrosingopine, followed by WST-1 assay at 0 h, 24 h, 48 h, and 72 h, respectively. ^∗∗^*P* < 0.01, 0 μM syrosingopine treatment group *vs*. 1 μM, 10 μM, or 100 μM syrosingopine treatment group at the indicated time points, respectively. **(B)** The effect of syrosingopine on the lactate levels in LX2 cells. LX2 cells were treated with 10 μM syrosingopine or DMSO for 24 h and were lysed for the determination of intracellular lactate concentrations. ^∗∗^*P* < 0.01, syrosingopine treatment group (Syro) *vs*. DMSO control group (DMSO). **(C)** The effect of syrosingopine on the morphology and organelle substructure of LX2 cells. LX2 cells were treated with 10 μM syrosingopine or DMSO for 24 h, and subjected to examinations under a bright field microscope (200×) **(a)** and transmission electron microscope (DMSO: 6000×, 12,000×; Syro: 8000×, 20,000 × ) **(b),** respectively. Autophagosomes are indicated with red arrows (20,000×). **(D)** The effect of syrosingopine on mRNA levels of key genes involved in liver fibrosis *in vitro*. LX2 cells were treated with 10 μM syrosingopine or DMSO for 36 h, total RNA was isolated and subjected to touchdown qPCR analysis, ^∗^*P* < 0.05, ^∗∗^*P* < 0.01, syrosingopine treatment group (Syro) *vs*. DMSO control group (DMSO). **(E)** The effect of syrosingopine on protein levels of key genes involved in liver fibrosis *in vitro*. LX2 cells were treated with 10 μM syrosingopine or DMSO for 72 h, and total protein was prepared and subjected to western blot analysis. **(F)** The effect of syrosingopine on the body weight of normal mice for eight weeks. ^∗^*P* < 0.05, ^∗∗^*P* < 0.01, syrosingopine treatment group (Syro) *vs*. DMSO control group (NC) at the indicated time points. **(G)** The effect of syrosingopine on the body weight of the mice with liver fibrosis for five weeks. ^∗^*P* < 0.05, ^∗∗^*P* < 0.01, syrosingopine treatment liver fibrosis group (Fib + Syro) *vs*. DMSO control liver fibrosis group (Fib) at the indicated time points. **(H)** The histologic evaluation and immunohistochemical staining of the liver tissue treated with syrosingopine. The retrieved liver masses were fixed with PBS-buffered formalin and paraffin-embedded. The tissues were sectioned and subjected to hematoxylin and eosin staining **(a)**, Masson trichrome staining **(b)**, Sirius red staining **(c)**, and immunohistochemical staining with antibodies against α-SMA, IFN-γ, COLⅠ, and FN1 **(d)**. The staining results were recorded under a bright field microscope (100×; 400×). Representative results are shown.

Next, we found that by qPCR analysis the mRNA levels of *α-SMA*, *IFN-γ*, *Mmp1*, and *Mmp2* were significantly up-regulated upon syrosingopine (Fig. 1D). Western blotting results also showed that the expression of IFN-γ, α-SMA, and COLⅠ was elevated at the protein level upon syrosingopine treatment (Fig. 1E). Interestingly, transforming growth factor beta, one of the most prominent profibrotic factors,^4^ was down-regulated by syrosingopine treatment. Collectively, these results strongly suggest that syrosingopine may activate HSCs *in vitro*.

In our *in vivo* study, we found that intraperitoneal injection of syrosingopine significantly reduced the body weight of normal mice as early as the fourth week (Fig. 1F). Similar results were also found in the mice with liver fibrosis so the syrosingopine experiment groups were terminated at the fifth week to avoid animal fatality (Fig. 1G). Hematoxylin and eosin staining showed the disorganization arrangement of hepatocytes and the disappearance of the nucleus of liver tissues in the syrosingopine treatment group, compared with those in the control group. The liver tissue completely lost its normal histologic arrangement with a large number of pseudolobules and a large number of liver cells underwent balloon-like transformation of liver tissues both in the syrosingopine-treated liver fibrosis group and the liver fibrosis group. In fact, more fibrotic pseudolobules were found in the fibrosis mice treated with syrosingopine, suggesting that syrosingopine may exacerbate liver fibrosis (Fig. 1H, panel a).

The results from Masson trichrome staining and Sirius red staining further confirmed that syrosingopine treatment increased collagen deposition in liver tissue, especially in the fibrotic liver tissue (Fig. 1H, panel b, c). Immunohistochemistry staining also revealed the high expression levels of α-SMA, IFN-γ, COLⅠ, and FN1 in the liver tissue after syrosingopine administration (Fig. 1H, panel d). Taken together, these results suggest that syrosingopine may cause or exacerbate liver fibrosis *in vivo*.

In conclusion, we demonstrate that the antihypertensive drug and lactate transporter (MCT1/4) inhibitor syrosingopine effectively activates the HSCs by inhibiting lactate efflux, which subsequently promotes the development and progression of liver fibrosis. These findings suggest that restoration or facilitation of lactate efflux may serve as a potential therapeutic strategy to treat or alleviate hepatic fibrosis.

## Author contributions

JF and TCH conceived and designed the study. MG performed the experiments and collected the data. MG and YG performed statistical analysis. JZ, XD, AL, and AH participated in the experiments; provided essential experimental materials, and assisted in qPCR data analysis and interpretations. MG and JF drafted and revised the manuscript. All authors reviewed and approved the final manuscript.

## Conflict of interests

The authors declare no conflict of interests.

## Funding

The reported study was supported in part by research grants from the 10.13039/501100001809National Natural Science Foundation of China (No. 82102696 to JMF) and the 10.13039/100000002National Institutes of Health (USA) (No. CA226303 to TCH). TCH was also supported by the Mabel Green Myers Research Endowment Fund and The 10.13039/100007234University of Chicago Orthopaedics Alumni Fund. Funding sources were not involved in the study design, in the collection, analysis, and interpretation of data, in the writing of the report, and in the decision to submit the paper for publication.

## Data availability

All datasets generated for this study are included in the manuscript and/or the Supplementary Material. Any further inquiries about data and resource availability can be directed to the corresponding authors.
