# Supplementary material for: Syrosingopine, an anti-hypertensive drug and lactate transporter (MCT1/4) inhibitor, activates hepatic stellate cells and exacerbates liver fibrosis in a mouse model
Source: Genes Dis. 2023 Nov 18;11(4):101169. doi: 10.1016/j.gendis.2023.101169 (PMC10909599; doi:10.1016/j.gendis.2023.101169)
Supplement: Multimedia component 1 [file mmc1.docx]

**Syrosingopine, an anti-hypertensive drug and lactate transporter (MCT1/MCT4) inhibitor, activates hepatic stellate cells and exacerbates liver fibrosis in a mouse model**

Meichun Guo^1^, Yannian Gou^1^, Xiangyu Dong^1^, Jiamin Zhong^1,2^, Aohua Li^1^, Ailing Hao^1^, Tong-Chuan He^2^ and Jiaming Fan^1^*

^1^ Ministry of Education Key Laboratory of Diagnostic Medicine, and the School of Laboratory Diagnostic Medicine, Chongqing Medical University, Chongqing, China

^2^ Molecular Oncology Laboratory, Department of Orthopaedic Surgery and Rehabilitation Medicine, The University of Chicago Medical Center, Chicago, IL 60637, USA

* Corresponding authors.

CORRESPONDENCES

Jiaming Fan, MD, PhD

Ministry of Education Key Laboratory of Diagnostic Medicine

Department of Clinical Biochemistry

School of Laboratory Medicine

Chongqing Medical University

Chongqing, 400016, China

Tel. +86 23 68485240

Email: [fanjiaming1988@cqmu.edu.cn](mailto:fanjiaming1988@cqmu.edu.cn)

**SUPPLEMENTAL MATERIALS**

**Materials and Method**

**Cell culture and chemicals**

The human immortalized hepatic stellate cells (LX2) were cultured in DMEM supplemented with 10% fetal bovine serum (Lonsera, Cat: S711-0015, Uruguay) containing 100 units of penicillin and 100 µg/mL of streptomycin in 5% CO_2_ incubators at 37°C ^1-3^. Syrosingopine (cat# S9907, CAS 84-36-6) was purchased from Selleck Chemicals. Syrosingopine was dissolved in dimethylsulfoxide (DMSO) to prepare for a stock solution of 10mM and was kept at -80°C. Unless indicated otherwise, all other chemicals were purchased from Sigma-Aldrich, ThermoFisher Scientific, or Solarbio.

**WST-1 cell proliferation assay**

Exponentially growing cells were seeded in 96-well plates (2,000 cells/well) and treated by different syrosingopine concentrations. At the indicated time points, the Premixed WST-1 Reagent (Clontech, Mountain View, CA) was added, followed by incubating at 37°C for 120min and reading absorbance at 450nm using a microplate reader (Biotek, EON, USA) as described^1,3-6^.

**Transmission electron microscope (TEM) analysis**

Approximately 2×10^6^ cells were collected by centrifugation at 1,200 rpm for 10min. The cell pellets were fixed with 2.5% glutaraldehyde and stored at 4°C. The cell pellets were washed with 0.1M sodium cacodylate and further fixed with 4% osmium tetroxide for 1h, followed by serial dehydration in ascending concentrations of acetone (from 35% to 100%). The samples were infiltrated with a mixture of acetone and resin at the ratio of 1:1 for 1 h, 1:2 for 2h and 100% resin overnight as reported ^7^. Finally, the samples were polymerized at 60°C for 36h prior sectioning to prepare the 90nm thick sections using the ultra-microtome Leica EM UC7 (Leica, Germany). The ultrathin sections were stained with lead citrate/uranyl acetate and analyzed under a transmission electron microscope (Hitachi 7500, Japan).

**Determination of cellular lactate content**

Prepared cells (5×10^6^ cells) were lysed with 500μl of 1-2% triton x 100 for 30min in PBS. According to the protocol of the lactate concentration determination kit (A019-2-1, JianCheng, NanJing, China), the working buffer and coloration reagent were mixed, and cell lysate was added to the reaction solution and incubated at 37˚C for 10min^8^. Absorbance was measured at 530nm using a microplate reader (Biotek, EON, USA).

**RNA isolation and touchdown-quantitative real-time PCR (TqPCR)**

Total RNA was isolated by using the TRIZOL Reagent (Invitrogen, China), and subjected to reverse transcription using hexamer and M-MuLV reverse transcriptase (New England Biolabs, Ipswich, MA). The cDNA products were used as PCR templates. Gene-specific PCR primers were designed by using Primer3 program (**Table S1**). TqPCR was carried out by using 2x SYBR Green qPCR Master Mix (Bimake, Shanghai, China) on the CFX-Connect unit (Bio-Rad Laboratories, Hercules, CA) as described^9^. All TqPCR reactions were done in triplicate. *Gapdh* was used as a reference gene. Quantification of gene expression was carried out by using the 2^-ΔΔCq^ method as described^10,11^.

**Western blotting analysis**

Western blotting assay was carried out as previously described ^2^. The primary antibodies against β-ACTIN (1:5000-1:50000 dilution; Proteintech; Cat# 60009-1-Ig), α-SMA(1:1000 dilution; Bimake; Cat# A5550), Col1a1 (1:500-1:1000 dilution; Wanleibio; Cat# WL0088), TGF-β1 (1:500-1:1000 dilution; Wanleibio; Cat# WL02193), and IFN-γ(1:500-1:1000 dilution; Wanleibio; Cat# WL02440), the secondary antibodies (1:5000 dilution; ZSGB-BIG; Peroxidase-Conjugated Rabbit anti-Goat IgG or Peroxidase-Conjugated Goat anti-Mouse IgG, Cat# ZB-2306 or 2305). Immune-reactive signals were visualized with the Enhanced Chemiluminescence (ECL) kit (Millipore, USA) and recorded by using the Bio-Rad ChemiDoc Imager (Hercules, CA).

**Mouse model of liver fibrosis and *in vivo* experiments**

The use and care of mice were approved by the Research Ethics and Regulations Committee of Chongqing Medical University, Chongqing, China. The animals were obtained from and housed in the Experimental Animal Research Center of Chongqing Medical University. All experimental procedures followed the approved guidelines. For the model in **Figure 1F,** C57BL/6J mice (male, 5-week-old) were randomly divided into 2 groups, with 5 mice in each group. For the syrosingopine treated group (Syro), mice were intraperitoneally injected with 7.5mg/Kg body weight (g.b.w.) of syrosingopine every other day for 8 weeks. Equal volume of DMSO was used at the same frequency to treat the normal group (NC). For the model in **Figure 1G,** C57BL/6J mice (male, 5-week-old) were randomly divided into 2 groups, with 5 mice in each group, and were intraperitoneally injected with 2.0μl/g body weight (g.b.w.) of 20% CCl_4_ solution (wt/vol, in olive oil) twice a week. For the syrosingopine treated liver fibrosis group (Fib+Syro), mice were intraperitoneally injected with 7.5mg/Kg body weight (g.b.w.) of syrosingopine for 5 weeks. Equal volume of DMSO was used at the same frequency to treat the liver fibrosis group (Fib). The dynamic body weight of mice was recorded every week.

**Histological staining**

Retrieved masses were fixed in 10% formalin, and subjected to paraffin embedding, followed by sectioning. Serial sections were subjected to H & E staining, Sirius Red staining (Picro Sirius Red solution, G1471, Solarbio) and Masson’s trichrome staining (Masson's Trichrome Stain Kit, G1340, Solarbio, China)^3,12,13^. IHC staining was carried out by using the IHC staining kit (SP Kit, SP-9001, ZSGB-Bio, China) as described^14,15^. Briefly, the sections were deparaffinized and subjected to immunostaining with the primary antibodies against α-SMA (1:1000 dilution; Bimake; Cat# A5550) and IFN-γ (1:500-1:1000 dilution; Wanleibio; Cat# WL02440). The Biotin labeled goat anti-mouse IgG (SP Kit, SP-9000, ZSGB-Bio, China) or goat anti-rabbit IgG (SP Kit, SP-9001, ZSGB-Bio, China) and HRP-conjugated streptavidin were used to visualize the presence of the proteins of interest. Hematoxylin was used to stain the nuclei. Sections incubated without primary antibodies were used negative controls. Staining results were recorded under a bright field microscope (magnification, x100 or x400). Each assay condition was done in triplicate.

**Statistical analysis**

All experiments performed at least three times and/or repeated in three independent batches. Data were analyzed using GraphPad Prism 8 and presented as mean ± standard deviation (SD). Statistical significance was confirmed by one-way analysis of variance and the student’s t-test for the comparisons between groups. A value of P < 0.05 was considered statistically significant.


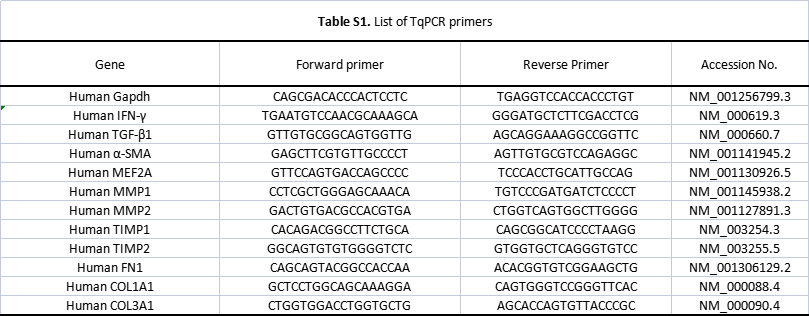


**REFERENCES**

1. Cui J, Zhang W, Huang E, et al. BMP9-induced osteoblastic differentiation requires functional Notch signaling in mesenchymal stem cells. *Lab Invest.* 2019;99(1):58-71.

2. Wang H, Cao Y, Shu L, et al. Long non-coding RNA (lncRNA) H19 induces hepatic steatosis through activating MLXIPL and mTORC1 networks in hepatocytes. *J Cell Mol Med.* 2020;24(2):1399-1412.

3. Zhong J, Wang H, Yang K, et al. Reversibly immortalized keratinocytes (iKera) facilitate re-epithelization and skin wound healing: Potential applications in cell-based skin tissue engineering. *Bioact Mater.* 2022;9:523-540.

4. Deng Y, Zhang J, Wang Z, et al. Antibiotic monensin synergizes with EGFR inhibitors and oxaliplatin to suppress the proliferation of human ovarian cancer cells. *Sci Rep.* 2015;5:17523.

5. Yu X, Liu F, Zeng L, et al. Niclosamide Exhibits Potent Anticancer Activity and Synergizes with Sorafenib in Human Renal Cell Cancer Cells. *Cell Physiol Biochem.* 2018;47(3):957-971.

6. Wang X, Wu X, Zhang Z, et al. Monensin inhibits cell proliferation and tumor growth of chemo-resistant pancreatic cancer cells by targeting the EGFR signaling pathway. *Sci Rep.* 2018;8(1):17914.

7. Law JX, Chowdhury SR, Saim AB, Idrus RBH. Platelet-rich plasma with keratinocytes and fibroblasts enhance healing of full-thickness wounds. *Journal of tissue viability.* 2017;26(3):208-215.

8. Wang T, Chen K, Yao W, et al. Acetylation of lactate dehydrogenase B drives NAFLD progression by impairing lactate clearance. *Journal of hepatology.* 2021;74(5):1038-1052.

9. Zhang Q, Wang J, Deng F, et al. TqPCR: A Touchdown qPCR Assay with Significantly Improved Detection Sensitivity and Amplification Efficiency of SYBR Green qPCR. *PLoS One.* 2015;10(7):e0132666.

10. Liao J, Wei Q, Fan J, et al. Characterization of retroviral infectivity and superinfection resistance during retrovirus-mediated transduction of mammalian cells. *Gene Ther.* 2017;24(6):333-341.

11. Shu Y, Wu K, Zeng Z, et al. A Simplified System to Express Circularized Inhibitors of miRNA for Stable and Potent Suppression of miRNA Functions. *Mol Ther Nucleic Acids.* 2018;13:556-567.

12. Zhang Z, Liu J, Zeng Z, et al. lncRNA Rmst acts as an important mediator of BMP9-induced osteogenic differentiation of mesenchymal stem cells (MSCs) by antagonizing Notch-targeting microRNAs. *Aging.* 2019;11(24):12476-12496.

13. Zhao C, Qazvini NT, Sadati M, et al. A pH-Triggered, Self-Assembled, and Bioprintable Hybrid Hydrogel Scaffold for Mesenchymal Stem Cell Based Bone Tissue Engineering. *ACS Appl Mater Interfaces.* 2019;11(9):8749-8762.

14. Hu N, Jiang D, Huang E, et al. BMP9-regulated angiogenic signaling plays an important role in the osteogenic differentiation of mesenchymal progenitor cells. *J Cell Sci.* 2013;126(Pt 2):532-541.

15. Huang X, Wang F, Zhao C, et al. Dentinogenesis and Tooth-Alveolar Bone Complex Defects in BMP9/GDF2 Knockout Mice. *Stem Cells Dev.* 2019;28(10):683-694.
