# Supplementary material for: Syrosingopine, an anti-hypertensive drug and lactate transporter (MCT1/4) inhibitor, activates hepatic stellate cells and exacerbates liver fibrosis in a mouse model
Source: Genes Dis. 2023 Nov 18;11(4):101169. doi: 10.1016/j.gendis.2023.101169 (PMC10909599; doi:10.1016/j.gendis.2023.101169)
Supplement: Multimedia component 2 [file mmc2.docx]

**Syrosingopine, an anti-hypertensive drug and lactate transporter (MCT1/MCT4) inhibitor, activates hepatic stellate cells and exacerbates liver fibrosis in a mouse model**

**Running title: Potential hepatotoxicity of syrosingopine**

Meichun Guo^1^, Yannian Gou^1^, Xiangyu Dong^1^, Jiamin Zhong^1,2^, Aohua Li^1^, Ailing Hao^1^, Tong-Chuan He^2^ and Jiaming Fan^1^*

^1^ Ministry of Education Key Laboratory of Diagnostic Medicine, and the School of Laboratory Diagnostic Medicine, Chongqing Medical University, Chongqing, China

^2^ Molecular Oncology Laboratory, Department of Orthopaedic Surgery and Rehabilitation Medicine, The University of Chicago Medical Center, Chicago, IL 60637, USA

* Corresponding authors.

CORRESPONDENCES

Jiaming Fan, MD, PhD

Ministry of Education Key Laboratory of Diagnostic Medicine

Department of Clinical Biochemistry

School of Laboratory Medicine

Chongqing Medical University

Chongqing, 400016, China

Tel. +86 23 68485240

Email: [fanjiaming1988@cqmu.edu.cn](mailto:fanjiaming1988@cqmu.edu.cn)

Syrosingopine is an anti-hypertensive drug and can cause high intracellular lactate levels and thereby end-product inhibition of lactate dehydrogenase by inhibiting the Lactate Transporters MCT1 and MCT4. Previous studies have shown that syrosingopine plays an essential role in the process of glycolytic blockade, ATP depletion and cell death in cancer by due to high intracellular levels of lactate^1,2^. Liver is the largest digestive gland in the human body and plays a crucial role in regulating energy metabolism, as well as serving as an important site for drug metabolism. Liver fibrosis is one of the most common pathological changes in the liver, which is a dynamic, highly complex molecular and cellular process leading to the excess accumulation of extracellular matrix (ECM) components sustained by heterogeneous population of hepatic myofibroblasts (MFs). The fibrosis usually follows chronic and long-term liver injury, which may progress to hepatic cirrhosis, hepatocellular carcinoma and liver failure^3^. The major driver of liver fibrogenesis is the activated hepatic stellate cells (HSCs), which are also the major cellular sources of excessive matrix protein secretion^4^. ^1,2^In this study, we investigated the effects of syrosingopine on HSCs, in the progression of liver fibrosis.

Optimal concentration of syrosingopine was first determined by treating human immortalized hepatic stellate (LX2) cells, and 10μM syrosingopine was used for further studies below **(Fig 1A)**. Syrosingopine was shown to significantly increase the lactate level in LX2 cells **(Fig 1B)**. Furthermore, the treated LX2 cells formed numerous dendrites, indicating that the hepatic stellate cells were activated and transformed into fibroblast-like cells **(Fig 1C, panel a).** During the process of HSC activation, intracellular energy-homeostasis is dysregulated, which is manifested by autophagy and endoplasmic reticulum stress^4^. We found in the LX2 cells treated with syrosingopine, the endoplasmic reticulum pool was slightly expanded, and protein synthesis decreased less, while the increased autophagy was apparent under transmission electron microscopy (TEM) **(Fig 1C, panel b)**.

Next, we found that by qPCR analysis the mRNA levels of *α-SMA, INF-γ, Mmp1* and *Mmp2* were significantly up-regulated upon syrosingopine **(Fig 1D)**. Western blotting results also showed that the expression of INF-γ, α-SMA and COLⅠwas elevated at protein levels upon syrosingopine treatment **(Fig 1E)**. Interestingly, transforming growth factor beta (TGF-β1), one of the most prominent profibrotic factors^4^, was down regulated by syrosingopine treatment. Collectively, these results strongly suggest that syrosingopine may activate HSCs *in vitro*.

In our *in vivo* study, we found that intraperitoneal injection of syrosingopine significantly reduced the body weight of normal mice at as early as week 4 **(Fig 1F).** Similar results were also found in the mice with liver fibrosis so the syrosingopine experiment groups were terminated at week 5 to avoid animal fatality **(Fig 1G)**. H & E staining showed the disorganization arrangement of hepatocytes and disappearance of nucleus of liver tissues in the syrosingopine treatment group, compared with that in the control group. The liver tissue completely lost its normal histologic arrangement with a large number of pseudolobules and a large number of liver cells underwent balloon-like transformation of liver tissues both in the syrosingopine treated liver fibrosis group and the liver fibrosis group. In fact, more fibrotic pseudolobules were found in the fibrosis mice treated with syrosingopine group, suggesting that syrosingopine may exacerbate liver fibrosis **(Fig 1H, panel a)**.

The results from Masson’ trichrome staining and Sirius Red staining further confirmed that syrosingopine treatment increased collagen deposition in liver tissue, especially in the fibrotic liver tissue **(Fig 1H, panel b and c)**. Immunohistochemistry (IHC) staining also revealed the high expression levels of α-SMA, INF-γ, COLⅠand FN1 in the liver tissue after syrosingopine administration **(Fig 1H, panel d)**. Taken together, these results suggest that syrosingopine may cause and/or exacerbate liver fibrosis *in vivo*.

In conclusion, we demonstrate that the antihypertensive drug and lactate transporter (MCT1/MCT4) inhibitor syrosingopine effectively activates the HSCs by inhibiting the lactate efflux, which subsequently promote the development and progression of liver fibrosis. These findings suggest that restoration or facilitation of lactate efflux may serve as a potential therapeutic strategy to treat or alleviate hepatic fibrosis.

**Acknowledgments**

The reported study was supported in part by research grants from the National Natural Science Foundation of China (82102696, JMF), and the National Institutes of Health (CA226303 to TCH). TCH was also supported by the Mabel Green Myers Research Endowment Fund and The University of Chicago Orthopaedics Alumni Fund. Funding sources were not involved in the study design, in the collection, analysis and interpretation of data, in the writing of the report, and in the decision to submit the paper for publication.

**Data Availability Statement**

All datasets generated for this study are included in the manuscript and/or the Supplementary Material. Any further inquiries about data and resource availability can be directed to the corresponding authors.

**Conflict of Interest**

The authors declare no conflict of interest.

**Author Contributions**

JF, TCH conceived and designed the study. MG performed the experiments and collected data. MG and YG performed statistical analysis. JZ, AL, AH participated in experiments; provided essential experimental materials; and assisted in qPCR data analysis and interpretations. MG, JF drafted and revised the manuscript. All authors reviewed and approved the final manuscript.

**Supplementary Information**

**Table S1**. List of qPCR Primers

**Authors and Affiliations**

Meichun Guo^1^, Yannian Gou^1^, Xiangyu Dong^1^, Jiamin Zhong^1,2^, Aohua Li^1^, Ailing Hao^1^, Tong-Chuan He^2^ and Jiaming Fan^1^*

^1^ Ministry of Education Key Laboratory of Diagnostic Medicine, and the School of Laboratory Diagnostic Medicine, Chongqing Medical University, Chongqing, China

^2^ Molecular Oncology Laboratory, Department of Orthopaedic Surgery and Rehabilitation Medicine, The University of Chicago Medical Center, Chicago, IL 60637, USA

* Corresponding author: Jiaming Fan, MD, PhD, Chongqing Medical University, Email: [fanjiaming1988@cqmu.edu.cn](mailto:fanjiaming1988@cqmu.edu.cn)

**REFERENCES**

1. Benjamin D, Colombi M, Hindupur SK, et al. Syrosingopine sensitizes cancer cells to killing by metformin. *Science advances.* 2016;2(12):e1601756.

2. Benjamin D, Robay D, Hindupur SK, et al. Dual Inhibition of the Lactate Transporters MCT1 and MCT4 Is Synthetic Lethal with Metformin due to NAD+ Depletion in Cancer Cells. *Cell reports.* 2018;25(11):3047-3058.e3044.

3. Parola M, Pinzani M. Liver fibrosis: Pathophysiology, pathogenetic targets and clinical issues. *Molecular aspects of medicine.* 2019;65:37-55.

4. Higashi T, Friedman SL, Hoshida Y. Hepatic stellate cells as key target in liver fibrosis. *Advanced drug delivery reviews.* 2017;121:27-42.

**Figure Legend**

**Figure 1.** Syrosingopine activates hepatic stellate cells and exacerbates liver fibrosis by upregulating the expression of α-SMA and INF-γ. **(A)** Optimal inhibitory concentration of syrosingopine in LX2 cells. LX2 cells were seeded in 96-well cell plates and treated with 0μM, 1μM, 10μM, 100μM syrosingopine, followed by WST-1 assay at 0h, 24h, 48h and 72h, respectively. “**” p<0.01, 0μM syrosingopine treatment group *vs* 1μM, 10μM, or 100μM syrosingopine treatment group at the indicated time points, respectively. **(B)** The effect of syrosingopine on the lactate levels in LX2 cells. LX2 cells were treated with 10μM syrosingopine or DMSO for 24h, and were lysed for the determination of intracellular lactate concentrations. “**” p<0.01, syrosingopine treatment group (Syro) *vs* DMSO control group (DMSO). **(C)** The effect of syrosingopine on the morphology and organelle substructure of LX2 cells. LX2 cells were treated with 10μM syrosingopine or DMSO for 24h, and subjected to examinations under bright field microscope (x200) **(*a*)** and transmission electron microscope (TEM) (DMSO x6,000, x12,000; Syro x8,000, x20,000) **(*b*),** respectively. Autophagosomes are indicated with red arrows (x20,000). **(D)** The effect of syrosingopine on mRNA levels of key genes involved in liver fibrosis *in vitro*. LX2 cells were treated with 10μM syrosingopine or DMSO for 36h, total RNA was isolated and subjected to TqPCR analysis, “*” p<0.05, “**” p<0.01, syrosingopine treatment group (Syro) *vs* DMSO control group (DMSO). **(E)** The effect of syrosingopine on protein levels of key genes involved in liver fibrosis *in vitro*. LX2 cells were treated with 10μM syrosingopine or DMSO for 72h, and total protein was prepared and subjected to Western blot analysis. **(F)** The effect of syrosingopine on the body weight of normal mice for 8 weeks. “*” p<0.05, “**” p<0.01, syrosingopine treatment group (Syro) *vs* DMSO control group (NC) at the indicated time points. **(G)** The effect of syrosingopine on the body weight of the mice with liver fibrosis for 5 weeks. “*” p<0.05, “**” p<0.01, syrosingopine treatment liver fibrosis group (Fib+Syro) *vs* DMSO control liver fibrosis group (Fib) at the indicated time points. **(H)** The histologic evaluation and immunohistochemical (IHC) staining of the liver tissue treated with syrosingopine. The retrieved liver masses were fixed with PBS-buffered formalin and paraffin- embedded. The tissues were sectioned and subjected to H & E staining **(*a*)**, Masson’ trichrome staining **(*b*)**, Sirius Red staining **(*c*)**, and immunohistochemical (IHC) staining with antibodies against α-SMA, INF-γ, COLⅠ, and FN1 **(*d*)**. The staining results were recorded under a bright field microscope (x100; x400). Representative results are shown.
